# Transaminase Elevations during Treatment of Chronic Hepatitis B Infection: Safety Considerations and Role in Achieving Functional Cure

**DOI:** 10.3390/v13050745

**Published:** 2021-04-23

**Authors:** Andrew Vaillant

**Affiliations:** Replicor Inc., 6100 Royalmount Avenue, Montreal, QC H4P 2R2, Canada; availlant@replicor.com

**Keywords:** transaminase flare, hepatitis B, functional cure, HBsAg

## Abstract

While current therapies for chronic HBV infection work well to control viremia and stop the progression of liver disease, the preferred outcome of therapy is the restoration of immune control of HBV infection, allowing therapy to be removed while maintaining effective suppression of infection and reversal of liver damage. This “functional cure” of chronic HBV infection is characterized by the absence of detectable viremia (HBV DNA) and antigenemia (HBsAg) and normal liver function and is the goal of new therapies in development. Functional cure requires removal of the ability of infected cells in the liver to produce the hepatitis B surface antigen. The increased observation of transaminase elevations with new therapies makes understanding the safety and therapeutic impact of these flares an increasingly important issue. This review examines the factors driving the appearance of transaminase elevations during therapy of chronic HBV infection and the interplay of these factors in assessing the safety and beneficial nature of these flares.

## 1. Introduction

Exposure to the hepatitis B virus (HBV) causes a predominantly hepatotropic infection, which in its acute state commonly causes liver inflammation and dysfunction and in its chronic state fibrosis, cirrhosis and hepatocellular carcinoma (HCC) [1,2,3]. While >80% of people infected with HBV achieve immune control and self-resolve their infection, reactivation of latent infection [4] in the livers of these individuals can still occur with immunosuppressive therapy [5,6]. Unfortunately, HBV infection remains chronic in more than 292 million people worldwide [7] and is responsible for 870,000 deaths annually [8]. This chronic infection results from (1) the inhibition of the immune response to HBV via the maintenance of abundant circulating HBsAg by the production of a large excess of non-infectious subviral particles (SVP) over virions [9], (2) the ability of the HBV genome to form a closed covalent circular DNA (cccDNA) “minichromosome” that can reside inside the nucleus of infected cells in an inactive “latent” state [10] and (3) the integration of HBV DNA into the chromosomes of liver cells [11,12], which progresses with continued chronic infection, becoming a significant source of SVP [9,13,14,15].

The current challenge in the treatment of chronic HBV infection is to restore immune control allowing suppression of viral infection to be maintained in the absence of therapy, thereby allowing liver damage to regress and the risk of developing hepatocellular carcinoma to decline. This “functional cure” of chronic HBV infection requires the reconstitution of immune control and is characterized by the absence of circulating HBV surface antigen (HBsAg) without therapy [16,17,18,19,20]. Since HBsAg is almost entirely derived from SVP [9], establishing a functional cure of HBV infection requires not only clearing SVP from the blood but removing the SVP producing capacity in the liver by the silencing of cccDNA and the removal of hepatocytes with integrated HBV DNA [14,15].

Since the clearance of hepatocytes with integrated HBV DNA from the liver is a critical milestone in achieving functional cure, the resulting damage in the liver is also expected to result in elevations in serum concentrations of liver enzymes. This creates a clinical challenge as liver enzyme flares are known to occur with a variety of other liver diseases where they are associated with hepatoxicity. Given the current focus on achieving a functional cure of HBV with new therapeutic agents, this review explores how liver enzyme flares during the treatment of chronic HBV infection affect liver function and the potential utility of these flares to predict positive therapeutic outcomes.

## 2. Distribution of HBV Infection in the Liver

**Figure 1:**
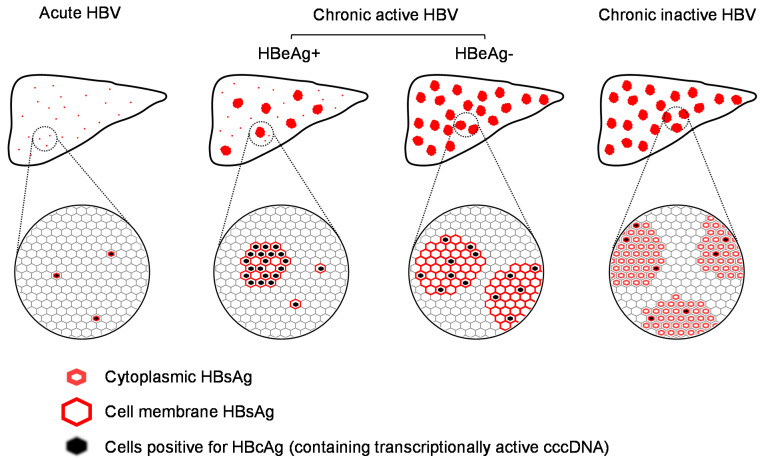
Distribution of infection in the liver during the course of HBV infection.

During acute (initial) infection, HBV infection appears infrequently in individual hepatocytes where HBsAg is located primarily in the cytoplasm [21]. These cells presumably also contain replicating virus. Transition to chronic infection (Figure 1) appears to be accompanied by translocation of HBsAg to the cell margins [21]. The number of cells in an initially infected liver is unknown, as symptoms of acute infection are considered to occur weeks to months after the initial infection event. In chronic infection, the bulk of infected cells in the liver are hepatocytes, but infection is also present to a lesser extent in the bile duct epithelium as well as endothelial and smooth muscle cells of hepatic blood vessels [22]. Scattered HBsAg positive hepatocytes are accompanied by HBcAg positivity approximately 70% of the time, and HBsAg is found both in the cytoplasm and at the cell margin [21,23]. With the transition from HBeAg positive to HBeAg negative infection, HBsAg distribution becomes mainly distributed in clusters of hepatocytes predominantly in the cytoplasm [24], in which HBcAg is less frequently detected (Figure 1). This clustered HBsAg+, HBcAg- pattern persists in inactive HBV infection with mostly cytoplasmic HBsAg localization [21,23]. These patterns are consistent with the progressive establishment of HBV infection in the liver during the natural history of HBV infection, with transition from individual hepatocytes producing virus and SVP to clusters of infected cells, which include hepatocytes containing only integrated HBV DNA and producing only SVP.

In chronic HBV infection, numerous studies have confirmed the heterogenous pattern of infected hepatocytes in chronic HBV infection (Figure 1) where infection is spread intermittently throughout the liver in patches comprised entirely of infected cells [21,23,24,25,26,27,28] or in sparsely infected regions [21,24,25,27,29,30]. In many instances, HBsAg expression is detected in cells devoid of HBV DNA or HBcAg [26,28,31,32,33] (Figure 1), which signals the presence of cells containing integrated HBV DNA, producing HBsAg efficiently (as SVP) but not HBV pgRNA (and HBV DNA) or other viral antigens [11]. Based on the best available biopsy estimates, the extent of liver infection (cells containing either cccDNA or integrated HBV DNA) in chronic HBV infection varies from 35% [34] to 70% [21]. While these studies demonstrate the expansion of HBV infection throughout the liver, they also demonstrate the preservation of normal functioning hepatic tissue throughout the natural history of HBV infection.

## 3. Impact of HBV Infection on Hepatocyte Function

Although HBV infection is not directly cytopathic, HBV infection of hepatocytes is followed by extensive metabolic reprogramming. These effects are summarized in Table 1 and consist of alterations in lipid metabolism leading to intracellular lipid and cholesterol accumulation [35,36,37,38,39,40,41,42,43,44,45], increased oxidative stress [39,46] and glucose metabolism [43,45], altered cell cycle regulation [44,47,48] and increased intracellular protein recycling [49,50,51]. HBV infection also sensitizes hepatocytes to apoptotic signaling [52,53]. These changes signal a reduction in the reservoir of normal liver infection driven by HBV replication in hepatocytes during acute and chronic HBV infection. These alterations may also be responsible in part for the reduced liver function observed with the expansion of HBV infection.

## 4. The Molecular Basis of Liver Enzyme Elevations in the Blood

**Figure 2:**
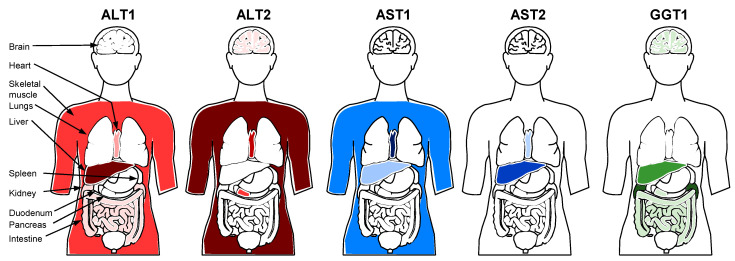
Relative tissue expression of ALT, AST and GGT isozymes in humans.

Alanine aminotransferase (ALT), also known as glutamic–pyruvic transaminase (GPT), is a cytosolic enzyme involved in gluconeogenesis that catalyzes the amination of α-ketoglutarate from alanine to produce pyruvate and glutamate. In humans, two distinct ALT isozymes are produced from distinct but related genes, ALT1 and the more recently characterized ALT2 [54], which both contribute to ALT activity detected in standard blood testing [55]. These two ALT isozymes have different cellular and tissue distributions (Figure 2), with ALT1 most enriched in the liver (ER and cytoplasm) and ALT2 most enriched in skeletal muscle (ER and mitochondria) and absent in the liver [55,56,57]. Both ALT isozymes are present in human plasma [55,57], but most of the catalytic ALT activity detected in normal plasma appears to derived from ALT2 [55].

Aspartate aminotransferase (AST), also known as glutamic oxaloacetic transaminase (GOT), catalyzes the reversible transfer of an amino group between aspartate and glutamate. There are two AST isozymes dervied from distinct genes which produce the ALT forms found either in the cytoplasm (AST1) or mitochondria (AST2) [58]. These isozymes also have different tissue distributions (Figure 2): AST1 is found predominantly in the heart [59] and striated muscle [56], and AST2 is found predominantly in the liver [60].

γ-glutamyl transferase (GGT) cleaves the γ-glutamyl moiety from glutathione and transfers this moiety to a variety of acceptors (including water to form glutamate). There are two GGT genes (GGT 1 and GGT 2), each producing GGT precursor protein; however, only the GGT1 precursor undergoes autocleavage to become catalytically active [61]. GGT (GGT1) is membrane associated and primarily found in the kidneys (in proximal renal tubules) but also in significant levels in the liver in the biliary epithelial cells with lower levels present in the canicular and sinusoidal surfaces of hepatocytes [62,63,64] (Figure 2).

In subjects with previously characterized viral hepatitis, elevations of ALT, AST and GGT in the blood are driven by release of these enzymes from cells in the liver. This release can occur via rupture of plasma membrane blebs formed in response to cellular stress or inflammation [65], from cellular necrosis [66] or due to immune-mediated cellular damage [67,68]. Chronic HBV infection is accompanied by elevations of serum levels of ALT and AST [69], the latter of which is used to guide the initiation of antiviral therapy under current treatment guidelines for chronic HBV infection [70,71,72]. Elevations in GGT have also been linked to the progression of liver disease [73], mortality [74] and clinical outcomes from treatment of HBV infection [75]. Elevations in these enzymes during the natural course of HBV infection can be accompanied by alterations in the reservoir of liver synthetic and secretory function [76] reflected in increased serum bilirubin, decreased serum albumin, decreased platelet count and/or increased INR (biochemical flare). Although circulating ALT is not only derived from the liver, it is uniformly present in hepatocytes, and elevations observed during HBV infection are considered the most sensitive marker for cellular damage to hepatocytes.

## 5. Transaminase Flares and the Hepatic Reservoir during Natural History of HBV Infection

Definitions of clinically significant transaminase elevations including flares range from >2–5x the upper limit of normal (ULN) [77,78,79,80] or >3x higher than baseline levels [77,79]. All of these definitions have been shown to be associated with impacts on disease, indicating that all transaminase elevations should be regarded as clinically significant during the natural history or treatment of chronic HBV infection. ALT elevations can occur throughout the natural history of chronic HBV but are rarely accompanied by biochemical flare or by hepatic decompensation, which is characterized by severe biochemical flares accompanied by jaundice, ascites or encephalopathy, or signs of severe anticoagulation (easy bruising or bleeding) [81,82]. Elevations are more frequently observed in HBeAg positive infection [83,84], where they are preceded by increased viremia [85] and followed by increased immune activity [81,86,87,88,89,90,91], indicating that these flares are driven by immune-mediated damage/clearance of infected hepatocytes. Biochemical flare, hepatic decompensation and additional extrahepatic manifestations [92] accompanying ALT elevation (in up to 30% of cases) are mainly observed in acute HBV infection and HBeAg positive chronic infection [86] when HBV DNA exceeds 1.55 × 10^9^ copies/mL (2.66 × 10^8^ IU/mL) [93]. ALT elevations during the natural course of HBV infection are typically followed by declines in HBV DNA and/or HBeAg seroconversion [82,94] concomitant with a reduction in the activity and hepatic burden of covalently closed circular DNA (cccDNA [95] and integrated HBV DNA [96].

**Figure 3:**
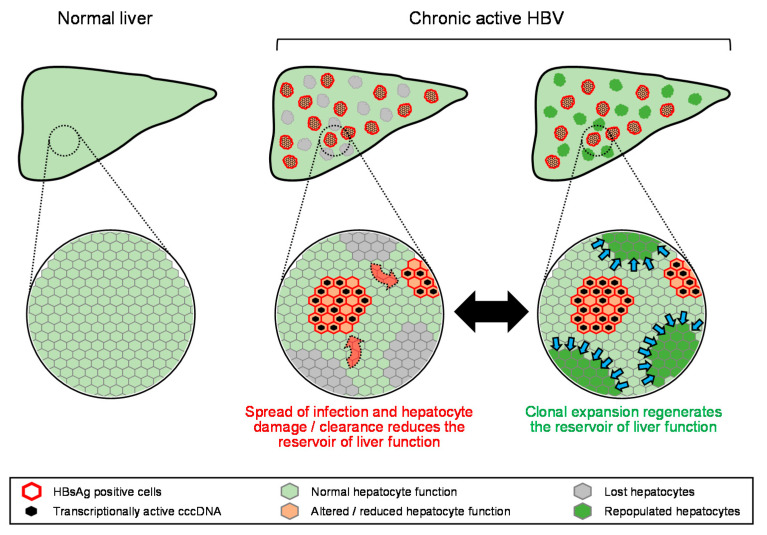
Model for the equilibrium between loss and regeneration of the hepatic reservoir during chronic HBV infection. Red arrows indicate expansion of HBV infection, blue arrows indicate regeneration of normal hepatocytes.

**Figure 4:**
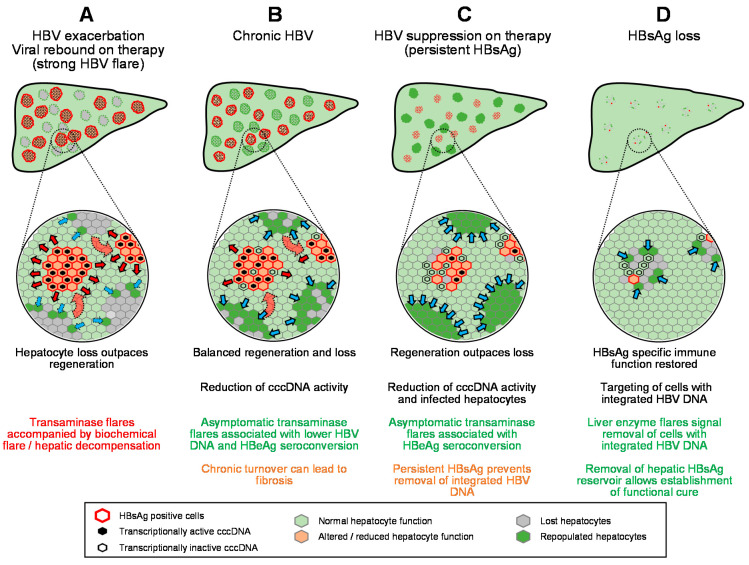
Model for the impact of transaminase flares on the hepatic reservoir in the absence or presence of antiviral therapy (**A–D**). HB sAg loss. Red arrows indicate expansion of HBV infection, blue arrows indicate regeneration of normal hepatocytes.

From the initial establishment of HBV infection in the liver and its spread during the evolution of chronic HBV infection, elevations in liver enzymes are driven by hepatocyte damage and loss from: (1) the increased apoptotic sensitivity in hepatocytes resulting from HBV infection, (2) inflammation driven by the innate immune response to HBV infection and (3) specific targeting of infected hepatocytes by the HBV specific adaptive immune response, both B-cell [97] and T-cell mediated [91]. These effects drive reduction in the reservoir of normal hepatic function, fueling biochemical flare and decompensation. However, the liver has well-established regenerative properties [98,99,100,101] and clonal expansion of normal hepatocytes has been characterized throughout HBV infection [102,103], although the extent and rate of this clonal expansion has yet to be determined [12]. Thus, the available reservoir of normal liver function during the progression of HBV is governed by the balance of the loss of normally functioning hepatocytes due to HBV infection and the clearance of HBV infected hepatocytes with the restoration of normally functioning hepatocytes due to regeneration (Figure 3). In this context, ALT elevation accompanied by biochemical flare and/or hepatic decompensation likely reflects the expansion of viral infection and the accompanying hepatocyte loss from inflammation and immune-mediated clearance at a rate faster than can be compensated for by regeneration (Figure 4A). Host-mediated transaminase flares likely represent aspects of immune control of infection being established with the net removal of infected hepatocytes not impacting liver function (Figure 4B). However, without complete immunological control being established, even slow chronic HBV reinfection and hepatocyte turnover can lead to the development of fibrosis, which will eventually erode the reservoir of normally functioning hepatocytes, leading to biochemical flare and hepatic decompensation.

## 6. Transaminase Flares during Therapy

The control of liver enzyme elevations is an important consideration during the management of chronic HBV infection as they can lead to hepatic decompensation, which is of special concern in patients with advanced fibrosis and cirrhosis who have only a fraction of the normal reservoir of hepatic function. As such, transaminase elevations, even if they may be beneficial, are generally recognized as an important indicator to initiate antiviral therapy to control liver dysfunction and the progression of liver disease. Current treatment guidelines with approved therapies [70,71,72] indicate the introduction of nucleos(t)ide analog inhibitors of the HBV reverse transcriptase (NUCs) only when chronic HBV infection is accompanied by HBV DNA > 2000 IU/mL and ALT elevation > ULN with the goal of providing long-term therapy for chronic suppression of infection. The use of pegylated interferon is also indicated for the treatment of chronic HBV infection with proper patient selection when the therapeutic goal is finite therapy leading to immunological control of infection (virologic response, as defined as normal ALT with HBV DNA < 2000 IU/mL).

### 6.1. Nucleos(t)ide Analogues

The guanine nucleoside analog entecavir (ETV), the adenosine nucleotide analog tenofovir disoproxil fumarate (TDF) and its more recent derivative tenofovir alafenamide (TAF) are the currently recommended NUC therapies [104] for the control of liver disease due to chronic HBV infection. These are bifunctional agents, having direct antiviral activity via inhibition of the HBV reverse transcriptase and indirect antiviral effect via the stimulation of innate immunity [105,106,107,108,109,110,111,112]. Transaminase flares observed during therapy with earlier generation NUCs such as lamivudine are typically associated with viral flare, and in some cases hepatic decompensation, as a result of the evolution of drug resistance to these early NUCs [113,114]. In these cases, a viral flare signals the expansion of infection in the liver and concomitant transaminase flares signal the removal of infected cells and/or cellular injury, resulting in net removal normal liver function at a pace greater than can be compensated for by regeneration (Figure 4A). The development of drug resistance to ETV and TDF/TAF is rare [115,116,117], and as a result, transaminase flares are less frequent with these NUCs but also not accompanied by biochemical flare or hepatic decompensation [79,114,118,119]. Flares during therapy with these later-generation NUCs are frequently associated with declines in HBV DNA, HBeAg seroconversion [79,119] and, in rarer instances, HBsAg loss [80,120], indicating an immunological basis for these flares. In these cases of transaminase flares during NUC-mediated HBV suppression, liver regeneration can match or outpace hepatocyte injury/infected hepatocyte loss (Figure 4C), preventing progression of fibrosis and hepatic decompensation.

### 6.2. Removal of NUC Therapy

Transaminase elevations often accompany removal of NUC therapy in HBeAg negative patients [121,122]. With older-generation NUCs such as lamivudine, these off-therapy flares were accompanied by biochemical flare and hepatic decompensation when HBV DNA levels were elevated [123,124]. Transaminase flares following withdrawal of TDF appear to occur earlier than withdrawal from ETV [125], but withdrawal from ETV or TDF is rarely accompanied by biochemical flare or hepatic decompensation [121,122,126,127]. Notwithstanding this, these host-mediated flares are not associated with sustained control of viremia, and re-treatment with NUCs is usually required [128].

### 6.3. Pegylated Interferon

Pegylated interferon (pegIFN) is a multifunctional immunotherapy that stimulates both innate [129,130] and adaptive immune responses [131,132] but also erodes T-cell function [131] as exposure increases. Transaminase flares are more frequently observed during pegIFN therapy than with NUCs and are driven either by viral flares which are more likely to be accompanied by biochemical flares and/or hepatic decompensation, or the host-mediated clearance of infected cells from the liver, which is followed by declines in HBV DNA [133,134] and has similar effects on the overall reservoir of liver function as observed with NUCs. Stronger host-mediated flares during pegIFN are associated with increased likelihood of HBV DNA decline, HBeAg seroconversion and increased rates of HBsAg loss and are not accompanied by biochemical flare or hepatic decompensation in non-cirrhotic patients [133,134,135,136,137,138,139].

### 6.4. Thymosin Alpha 1

Thymosin alpha 1 is synthetic form of a naturally occurring thymic peptide hormone that acts primarily as a T-cell agonist [140,141,142]. Treatment of chronic HBV infection with thymosin alpha 1 is also accompanied by transaminase flares, which either resolve during therapy [143] or appear and self-resolve after therapy [144] with no biochemical flare or hepatic decompensation and are associated with HBeAg seroconversion [143,144].

### 6.5. RNAi/Antisense

These oligonucleotide-based compounds are designed to target the cleavage of HBV mRNA via the RISC complex (in the case of RNAi) or via RNAse H (in the case of antisense). The specificity and activity of both these classes of oligonucleotides is strictly dependent on the perfect homology between the single-stranded (antisense) or double-stranded (RNAi) oligonucleotide and the target region of the mRNA. While in vivo data have been consistent with this mechanism, clinical studies with a variety of these compounds have indicated that off-target stimulation of innate immunity is playing a role in the clinical responses observed [9,145]. Limited data are available from ongoing trials with these compounds. However, the lipid-nanoparticle-formulated ARC-520 RNAi therapy was associated with mild transaminase flares during or after therapy, which self-resolved without biochemical flare or hepatic decompensation and in some cases were associated with declines in HBsAg [146]. Therapy with the GalNAc conjugated RNAi RG6346 (DCR-HBVS) was associated with transaminase flares in 3/6 participants at the 3 mg/kg dose in NUC-suppressed participants and accompanied by declines in HBV DNA and weaker declines in HBsAg [147].

### 6.6. Nucleic Acid Polymers

Nucleic acid polymers are broad spectrum antiviral agents [148] that selectively inhibit the assembly and secretion of HBV subviral particles without affecting secretion of HBeAg or virus [149,150]. Rapid and multilog HbsAg declines following NAP monotherapy in HBeAg positive infection are accompanied by strong, asymptomatic host-mediated transaminase flares, which are in turn accompanied by declines in HBV DNA, HBeAg seroconversion and HBsAg loss and seroconversion [151]. These flares are likely driven by rapid reduction in intrahepatic HBsAg, which restores innate immune function resulting in the clearance of infected cells and/or clearance of circulating HBsAg, which removes the exhaustion of the HBsAg-specific immune response, allowing the targeting of cells in the liver harboring both cccDNA and integrated HBV DNA [9]. In HBeAg negative co-infection with HDV, similar HBsAg declines are associated with much weaker host-mediated flares [152]. Introduction of pegIFN or thymosin alpha 1 greatly increases the strength and frequency of these host-mediated flares [151]. In the latest study of TDF, pegIFN and REP 2139 or REP 2165, 95% of participants experienced transaminase flares (ALT and AST) as well as GGT flares, all of which were host-mediated [153]. Recent analysis of the effects of these high rates of flares on therapeutic outcomes in this latest study indicated that transaminase flares occurring during HBsAg clearance were associated with partial or functional cure, while transaminase flares occurring in the presence of HBsAg were associated with viral rebound after removal of therapy [154]. In cases of transaminase flares with HBsAg loss, HBsAg-specific exhaustion is absent, suggesting that these transaminase flares are associated with removal of all HBsAg reactive hepatocytes, those with active cccDNA or with integrated HBV DNA (Figure 4D). Recent compassionate use of REP 2165 and TDF in a cirrhotic patient with HBV/HDV co-infection was accompanied by an early, host-mediated transaminase (ALT/AST) and GGT flare with no biochemical flare or hepatic decompensation [155].

### 6.7. Transaminase Flares during Therapy with Cirrhosis

**Figure 5:**
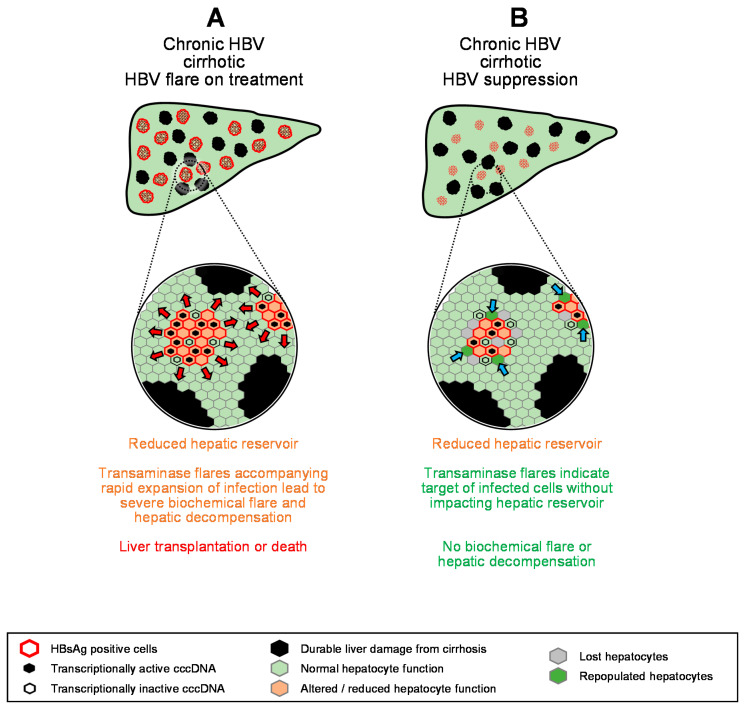
Model for the impact of transaminase flares on the hepatic reservoir in cirrhotic patients (**A**,**B**). Red arrows indicate expansion of HBV infection, blue arrows indicate regeneration of normal hepatocytes.

The benefit/risk of transaminase flares in the presence of cirrhosis is an important consideration as cirrhotic patients represent a small but significant proportion of patients of HBV monoinfection [156], with faster onset and greater prevalence in HBV/HDV co-infection [157,158]. Flares are beneficial in cirrhotic patients with antiviral response to non-pegylated INF, but in the absence of an antiviral response, flares are frequently accompanied by severe hepatic decompensation leading to death or liver transplantation [29,159]. With pegIFN, transaminase flares associated with hepatic decompensation were less frequent but still more commonly found in cirrhotic patients [133]. In these cases, additional depletion of the already diminished hepatic reservoir by reduction in functional hepatocytes from expansion of infection or increased hepatocyte loss drives severe hepatic decompensation (Figure 5A).

However, recent data have demonstrated that transaminase flares are beneficial in cirrhotic patients who achieved HBV suppression on NUC therapy, with increased HBeAg seroconversion, reduced incidence of ascites, esophageal varices, splenomegaly and death or liver transplantation and were not accompanied by biochemical flare or hepatic decompensation [160]. Moreover, combination therapy with TDF + pegIFN in patients with advanced cirrhosis with chronic HBV/HDV co-infection was also accompanied by host-derived transaminase flares, none of which were accompanied by biochemical flare or hepatic decompensation [161]. These more recent observations suggest that with the maintenance of effective HBV suppression, host-mediated transaminase flares do not impact the limited hepatic reservoir in cirrhotic patients (Figure 5B) and can be a means to safely achieve functional cure in cirrhotic patients.

## 7. Perspectives

With the overall goal of functional cure requiring the removal of hepatocytes with integrated HBV DNA and active cccDNA, transaminase flares are likely an essential milestone in this process and will be required for the achievement of HBsAg loss with currently approved therapies and therapies in development. The current clinical data indicate that with maintenance of viral suppression, transaminase flares appear to be universally host-mediated, do not negatively impact liver function and are associated with both improvement in virologic status and liver function and correlated with increased rates of functional cure of HBV, even with cirrhosis. As such, viral suppression during experimental therapies (such as NAPs) which yield both high rates of transaminase flares and functional cure should be accompanied by NUCs such as ETV, TDF or TAF to ensure safety during therapy.

However, higher-frequency monitoring of transaminases, GGT as well as markers of liver function should continue to be both essential data gathered during clinical trials for agents in HBV and be reported in detail to increase the available clinical database, especially during treatment of cirrhotic patients. Of important note here is GGT; although it is not considered as hepato-specific as ALT and AST, its elevation in the blood can signal effective targeting of infected cells in the bile duct epithelium and endothelial and smooth muscle cells of hepatic blood vessels. The reporting of changes in this liver enzyme should be included not only to indicate the targeting of non-hepatocyte reservoirs of HBV infection in the liver but also to better exclude a link between GGT elevations and other potential safety concerns.

**Figure 6:**
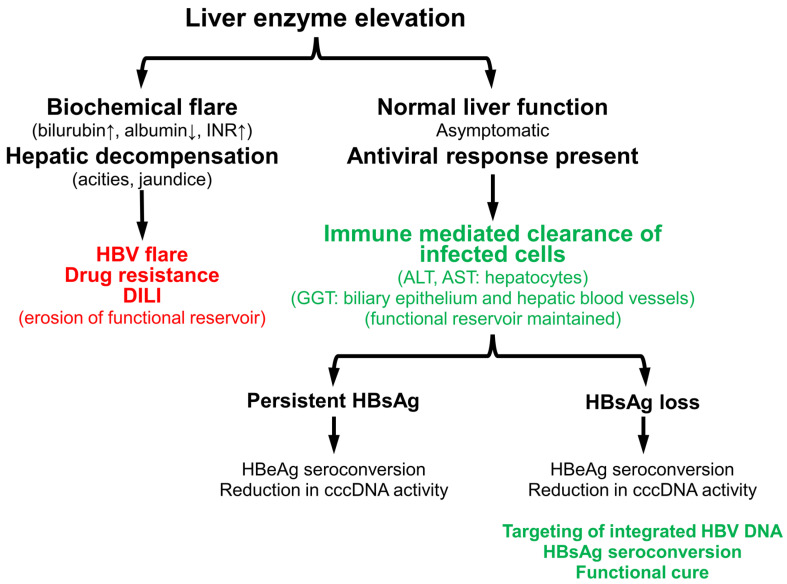
Identification and impact of detrimental and beneficial transaminase flares during the natural history or treatment of chronic HBV infection.

In the interim, the current clinical experience with transaminase flares indicates a common approach for interpreting these events with all antiviral agents (Figure 6). Transaminase flares driven by reduction of normal functioning hepatocytes due to increased viral activity in the liver or drug-induced liver injury outpace the regenerative capacity of the liver and lead to biochemical flare and/or hepatic decompensation. These flares are grounds for the introduction of HBV suppressive NUC (ETV/TDF/TAF) therapy or for dose reduction of immunotherapies or experimental antiviral agents. On the other hand, host-mediated flares are easily identified by the presence of an antiviral response and lack of biochemical flare or hepatic decompensation. These fares are highly correlated with HBV DNA reduction, HBeAg seroconversion and silencing/reduction of cccDNA and should not be grounds for halting therapy or dose reduction of experimental therapies in development. Strong flares with HBsAg loss may signal more efficient targeting of integrated HBV DNA and appear to be a marker for the establishment of functional cure with pegIFN and/or NAPs. This event may be required to establish high rates of functional cure in all patient populations, regardless of therapeutic approach. In the case of cirrhotic patients, the current clinical data suggest that transaminase flares are safe and well tolerated (even pegIFN-mediated) when effective HBV suppression (NUC therapy) is in place and that these flares are associated with improved virologic status. However, additional clinical data will be required to reinforce the observations of these recent studies.

## Figures and Tables

**Table 1:** Impact of HBV infection on hepatocyte function.

Parameter	Specific Change	Reference
Lipid metabolism	Downregulation of multiple apolipoproteins (A, B, C, E, F, H and M)	[35,36,37,38]
	Upregulation of fatty acid synthesis	[39]
	Increased cholesterol uptake and metabolism	[40]
	Increased lipogenesis, membrane biogenesis and intracellular lipid/cholesterol accumulation	[41,42,43,44,45]
Cellular Metabolism	Increased oxidative stress	[39]
	Downregulation of mitochondrial electron transport function and increased mitochondrial production of reactive oxygen species	[46]
	Upregulation of glycolysis and the Krebs cycle	[43,45]
Cell cycle	Transition from G_0_ to G_1_	[44,47,48]
Uptake/secretion	Upregulation of endocytosis and autophagy	[49,50]
	Upregulation of ER-associated protein degradation	[51]

## Data Availability

No new data were created or analyzed in this study. Data sharing is not applicable to this article.
